# Potential Treatment Options for Neuroblastoma with Polyphenols through Anti-Proliferative and Apoptotic Mechanisms

**DOI:** 10.3390/biom13030563

**Published:** 2023-03-20

**Authors:** Aisha Kafoud, Zoya Salahuddin, Raghad Sabaawi Ibrahim, Reem Al-Janahi, Alena Mazurakova, Peter Kubatka, Dietrich Büsselberg

**Affiliations:** 1 Weill Cornell Medicine-Qatar, Education City, Qatar Foundation, Doha P.O. Box 24144, Qatar; 2 Department of Anatomy, Jessenius Faculty of Medicine, Comenius University in Bratislava, 036 01 Martin, Slovakia; 3 Department of Medical Biology, Jessenius Faculty of Medicine, Comenius University in Bratislava, 036 01 Martin, Slovakia

**Keywords:** neuroblastoma, cancer, flavonoids, polyphenols, apoptosis

## Abstract

Neuroblastoma (NB) is an extracranial tumor of the peripheral nervous system arising from neural crest cells. It is the most common malignancy in infants and the most common extracranial solid tumor in children. The current treatment for high-risk NB involves chemotherapy and surgical resection followed by high-dose chemotherapy with autologous stem-cell rescue and radiation treatment. However, those with high-risk NB are susceptible to relapse and the long-term side effects of standard chemotherapy. Polyphenols, including the sub-class of flavonoids, contain more than one aromatic ring with hydroxyl groups. The literature demonstrates their utility in inducing the apoptosis of neuroblastoma cells, mostly in vitro and some in vivo. This review explores the use of various polyphenols outlined in primary studies, underlines the pathways involved in apoptotic activity, and discusses the dosage and delivery of these polyphenols. Primary studies were obtained from multiple databases with search the terms “neuroblastoma”, “flavonoid”, and “apoptosis”. The in vitro studies showed that polyphenols exert an apoptotic effect on several NB cell lines. These polyphenols include apigenin, genistein, didymin, rutin, quercetin, curcumin, resveratrol, butein, bisphenols, and various plant extracts. The mechanisms of the therapeutic effects include calpain-dependent pathways, receptor-mediated apoptosis, and, notably, and most frequently, mitochondrial apoptosis pathways, including the mitochondrial proteins Bax and Bcl-2. Overall, polyphenols demonstrate potency in decreasing NB proliferation and inducing apoptosis, indicating significant potential for further in vivo research.

## 1. Introduction

Neuroblastoma (NB) is an embryonal extracranial tumor of the peripheral nervous system derived from the neural crest. This cancer mainly affects children, with the median age of diagnosis being 17–18 months. Moreover, NB is the most common malignancy diagnosed within the first year of life [1]. It is likely to relapse and develop acquired drug resistance, posing a challenge in treatment [2]. The current treatment for high-risk NB involves chemotherapy and surgical resection followed by high-dose chemotherapy with autologous stem-cell rescue and radiation treatment [3]. However, those with high-risk NB are susceptible to relapses and the long-term side effects of standard chemotherapy [4]. The threat of multidrug resistance is also not uncommon, especially with the increasing intensity of therapy, which increases the likelihood of chemotherapeutic treatment failure in high-risk NB [5]. This necessitates the study of novel therapeutic agents, which would be less likely to result in adverse side effects. Many of these agents fall in the realm of natural dietary compounds, which several previous studies have demonstrated as having promising anti-cancer effects [6,7,8,9,10,11,12,13].

Natural polyphenols are organic compounds in plants that contain one phenol ring or more and exhibit anti-cancer potential [10,11,12]. Although they have the general structure of containing phenolic groups, polyphenols are a large and diverse class of molecules with many ubiquitous subgroups in plants, including fruits, teas, and vegetables. The main classes of polyphenols, as per one classification system, are phenolic acids (e.g., curcumin), phenolic alcohols, flavonoids, stilbenes (e.g., resveratrol), and lignans [14]. Polyphenols exert their anti-cancer properties in many ways, including but not limited to cell-cycle inhibition, the inhibition of proliferative enzymes, and apoptosis activation [13]. In general, polyphenols have low bioavailability for several reasons, such as their low water solubility, trouble with targeted delivery, and rapid elimination. However, polyphenols demonstrate significant biological effects; thus, a “low bioavailability/high bioactivity paradox” is highlighted in many studies [15].

Long-term polyphenol ingestion has been demonstrated to have positive and negative effects on human health and drug delivery. In vitro studies of green tea containing many polyphenols, such as EGCG, showed anti-cancer effects in breast, prostrate, liver, head, and neck cancers [16]. Green tea has also been linked to a lower risk of cognitive deterioration [17]. Polyphenols can slow the incidence rate of coronary heart disease by reducing platelet aggregation. Quercetin, for example, disrupts atherosclerotic plaque formation by inhibiting MMP1, which is involved in inflammation, atherosclerosis, and plaque formation [18,19]. Polyphenols also have anti-inflammatory and antioxidant properties and can help manage and prevent type 2 diabetes by protecting beta-pancreatic cells from glucose toxicity [20]. Clinical studies investigating polyphenol interaction with cancer cells often find that polyphenols have no adverse effect on normal cells [21].

**Figure 1:**
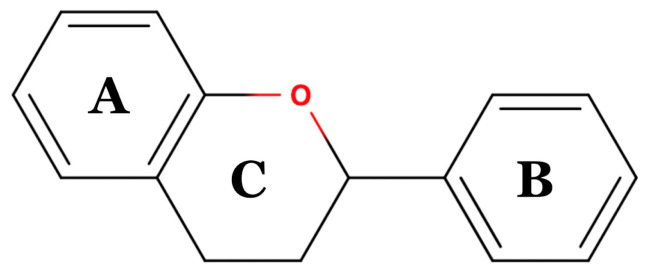
Basic skeletal structure of flavonoids. Created with MolView.com.

Flavonoids are naturally occurring chemicals and are members of the polyphenol family [22]. However, their structure distinguishes them from polyphenols; polyphenols have a more complicated structure than flavonoids. Almost all flavonoids have a C6–C3–C6 structure with two benzene rings, labeled A and B in Figure 1, connected by an oxygen-containing heterocyclic-pyrene ring (C), whereas polyphenols do not have a comparable structure [23].

There is firm support in the literature for the argument that flavonoids have a powerful anti-cancer effect. Flavonoids exert potent anti-neoplastic capacity via their modulation of reactive oxygen species (ROS) enzyme activity, support for cell-cycle arrest, induction of apoptosis, and lowering of proliferation [24]. Flavonoids are beneficial against a wide range of cancers, including NB. It is important to note that most studies on flavonoids, and polyphenols in general, are conducted with isolated compounds. However, complementary or synergistic interactions between natural compounds with similar anti-cancer mechanisms can enhance their overall curative and preventive properties and their bioavailability [10]. An understanding of how to apply flavonoids to NB in a way that has the best effect on the reduction in NB would be highly beneficial due to the various apoptotic and anti-proliferative pathways in NB cell lines that flavonoid treatments can exploit.

## 2. Study Methodology

The keywords “neuroblastoma”, “apoptosis” and “flavonoids” or “polyphenols” were used to obtain primary in vitro studies from multiple databases, including Google Scholar, MDPI, Springer, and PubMed. Studies were analyzed for relevant data, which included the identification of NB apoptosis followed by the application of compound, as well as insight into the apoptotic mechanisms. Studies concerning the neuroprotective, rather than the cytotoxic potential, of flavonoids were excluded. Twenty-six studies were included, from which four major apoptotic pathways were identified. Data were sorted into tables according to similarities in pathways, and shared apoptotic mechanisms were identified and mapped out in figures generated on BioRender.

## 3. Mechanisms of Apoptotic Action

### 3.1. Calpain-Dependent Apoptotic Pathway

**Figure 2:**
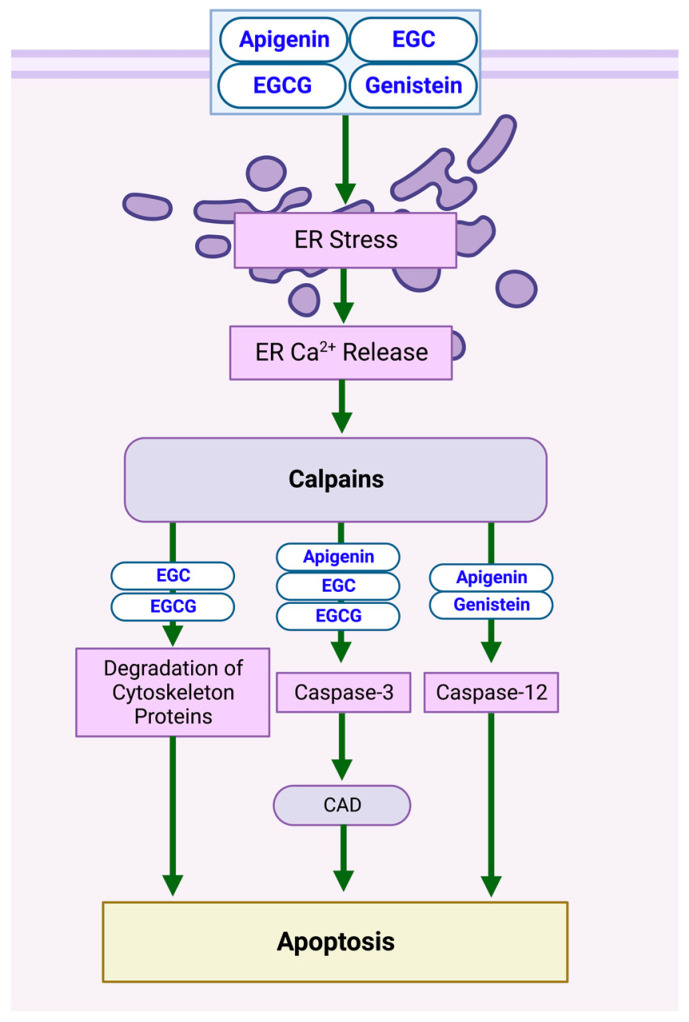
Schematic diagram demonstrating the apoptotic effects of flavonoids on NB cell lines via a calpain-dependent pathway. The induction of ER stress induces Ca^2+^ release at the ER membrane, triggering caspase and CAD release and degradation of cytoskeletal proteins. Specific biomarkers can be viewed in Table 1. Created with BioRender.com.

The release of calcium (Ca^2+^) from the endoplasmic reticulum (ER) leads to the activation of calpains. Calpain is a Ca^2+^-activated endo-protease involved in apoptotic mechanisms [25]. Exposure to the compounds listed in Table 1 activates proteolytic pathways involving calpain, leading to NB-cell apoptosis. Specifically, apigenin, epigallocatechin (EGC), epigallocatechin gallate (EGCG), and genistein trigger ER stress, thus increasing intracellular free Ca^2+^ and causing calpain activation at the ER membrane [26,27], resulting in the degradation of cytoskeletal proteins and the destabilization of cellular integrity in SH-SY5Y cells. Caspase release is also stimulated, with caspase-3 activating caspase-activated DNase (CAD), contributing to DNA fragmentation [8,28]. Caspase-12 is also activated when apigenin and genistein are applied, reinforcing their apoptotic effects [27]. Several studies concerning other cancer types reinforced the calpain–caspase apoptotic pathway, illustrated in Figure 2 [25,26,27,28,29].

### 3.2. Anti-Proliferative Pathways

**Figure 3:**
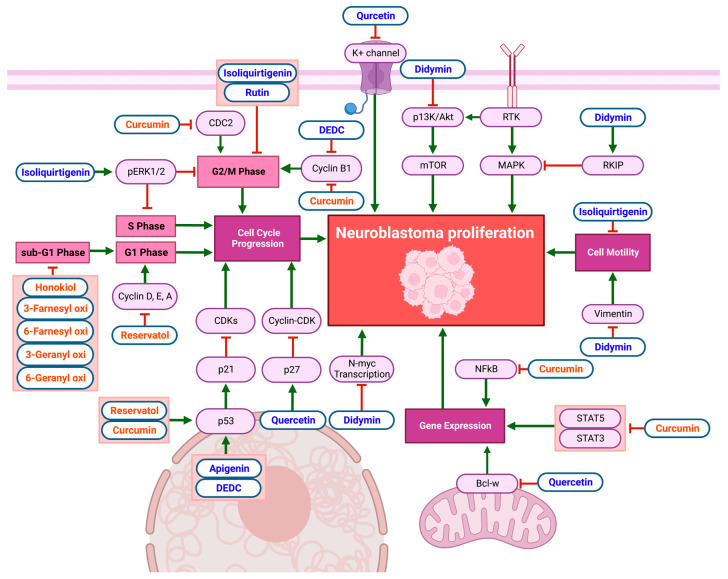
Schematic diagram demonstrating the anti-proliferative effects of flavonoids (blue) and non-flavonoid polyphenols (orange) on NB cell lines. The inhibition of cell-cycle progression, cell motility, and gene expression limits NB-cell proliferation. Compounds caused cell-cycle arrest in the S phase, sub-G1 phase, G1 phase, and G2/M phase. Specific biomarkers can be viewed in Table 2. Created with BioRender.com.

In vitro studies on the anti-cancer effects of flavonoids and non-flavonoid polyphenols in NB cell lines reveal remarkable anti-proliferative effects, as demonstrated in Figure 3 and Table 2. Quercetin exhibits anti-proliferative effects by increasing p27 mRNA expression, inhibiting the formation and activity of the cyclin–cyclin-dependent kinase (cyclin/CDK) complex, disrupting the cell cycle in NB. Quercetin also reduces B-cell lymphoma-w (Bcl-w) mRNA expression, which decreases tumor-gene expression and induces apoptosis in NB cells [30]. Quercetin can further inhibit NB-cell growth by blocking voltage-gated potassium (K^+^) channel activity [31]. Other flavonoids, such as apigenin and 2-(cis-1,2-dihydroxy-4-oxo-cyclohex-5-enyl)-5,7-dihydroxy-chromone (DEDC), increase p53 and p21 mRNA expression while decreasing cyclin-B1 expression in NB [32,33]. Didymin decreases the proliferation of NB cells via the downregulation of the phosphoinositide 3-kinase (PI3K) and Akt pathways, accompanied by reduced vimentin levels, indicating a decrease in cell motility. Furthermore, proto-oncogene N-Myc transcription was inhibited by didymin. Increased Raf-1 kinase inhibitor protein (RKIP) levels inhibit the mitogen-activated protein kinase (MAPK) pathway, also decreasing proliferation [34]. Isoliquiritigenin inhibits cell motility and increases the activation of extracellular regulated kinase 1/2 (pERK1/2), which inhibits NB-cell migration and proliferation while arresting the cell cycle in the S phase [35]. Isoliquiritigenin and rutin both enhance G2/M-phase arrest in NB [35,36].

Non-flavonoid polyphenols affect multiple cell lines through multiple pathways. Curcumin decreases CDC2 and cyclin-B1, resulting in NB-cell-cycle arrest in the G2/M phase [37]. Furthermore, it reduces NF-κB activator protein (AP-1) and STAT3 and STAT5 activation, suppressing gene transcription [38]. Honokiol inhibits NB-cell-cycle progression at the sub-G1 phase [39]. Resveratrol reduced Cyclin D1 levels in NB cells, causing cell-cycle arrest in the S phase [40]. Similarly, the treatment of NB cells with resveratrol resulted in a significant drop in pAkt, Cyclin D, E, A, and CDK2 levels and increased p53 and NF-κB, resulting in cell-cycle arrest in the S phase [41]. Resveratrol causes p21 levels to rise, which inhibits CDK levels and causes cell-cycle arrest in the G1, G2/M, and S phases in NB cell lines [42]. Prenyl hydroxy coumarin derivatives also have notable anti-proliferative effects on NB cell lines, inducing cell-cycle arrest in the sub-G1 phase, with no effect on normal lymphocytic cells [43].

In addition to isolated flavonoids and non-flavonoid polyphenolic compounds, recent research supports the potent anti-cancer effects of whole plants or plant extracts characterized by numerous phytochemicals acting synergistically or additively [44,45,46,47]. For example, a recent study by Morandi et al. (2021) demonstrated the capacity of olive-leaf extract (rich in phenolic compounds) to inhibit the proliferation of NB cells through cell arrest in the G0/G1 phase and the accumulation of cells in the sub-G0 phase, accompanied by the induction of apoptosis [48]. Numerous other recent studies highlight the anti-cancer potential of plant extracts; for example, the fruit extract of *Kigelia Africana*, a plant rich in flavonoids that are used in traditional African medicine, inhibited proliferation and other mechanisms associated with carcinogenesis in NB cells [49]. Interestingly, research results obtained by Roomi et al. (2013) suggested the therapeutic potential of a nutrient mixture of lysine, proline, ascorbic acid, and green-tea extracts for NB management through the inhibition of tumor growth and proliferation and the induction of apoptosis in neuroblastoma models in vitro and in vivo [50]. Indeed, green tea is rich in numerous phytochemicals, mainly catechins. Green-tea catechins, including ECG, EGCG, and EGC, are phytochemicals with strong anti-cancer effects [51,52].

**Table 2:** Results of in vitro studies of polyphenols’ anti-proliferative effects on NB cell lines *.

Compound	Cell Line	Incubation Period	Concentration(s)	Biomarker Changes and Effects		Reference
Flavonoids						
Apigenin	NUB-7 and LAN-5	24 h	10, 50, 100, 150, 200 µM IC_50_: 35 μM in NUB-7 IC_50_: 22 μM in LAN-5	↑ p53 ↑ p21^WAF−1/CIP−1^	↓ Proliferation	[33]
DEDC	SH-SY5Y	24 h	7.5 µg/mL	↑ p53 mRNA ↑ p21 mRNA ↓ Cyclin-B1		[32]
Didymin	CHLA-90 and SK-N-BE2 (p53-mutant) l + SMS-KCNR and LAN-5 (p53 wild-type)	24 h	50 μmol/L	↓ P13K ↓ Akt	↓ Proliferation	[34]
				↓ Vimentin	↓ Motility of tumor cells	
				↓ N-Myc transcription		
				↑ RKIP	↓ MAPK pathway ↓ Proliferation	
Isoliquiritigenin	SH-SY5Y	24 h	10–100 µM IC_50_: 25.4 µM	↑ pERK1/2	↓ Cell migration ↓ Proliferation ↑ S + G2/M-phase arrest	[35]
Rutin	LAN-5	24 h	0, 25, 50, 100 μM		↑ G2/M-phase arrest	[36]
Quercetin	Neuro2a (mouse cell line)	24 h	10, 20, 40, 80, 120 μM IC_50_: 40 µM	↑ p27	↓ Cyclin–CDK complex binding	[30]
				↓ Bcl-w	↓ Tumor-cell-gene expression	
Quercetin	Neuroblastoma X glioma NG 108-15 cells (mouse cell line)	48 h	10 µM, 20 µM IC_50_: 10 µM	↓ K^+^-channel activity	↓ Cell growth	[31]
**Non-Flavonoid Polyphenols**						
Curcumin	SK-N-SH	24 h	8, 16, 32 µM	↓ CDC2 ↓ Cyclin B1	↑ G2/M-phase arrest	[37]
Curcumin	GI-L-IN, HTLA-230, SH-SY5Y, LAN5, SK-NBE2c, and IMR-32	18–72 h	0.1–25 µM	↓ NFκβ activator protein (AP-1) ↓ STAT3, STAT5 activation	↓ Cell growth	[38]
Curcumin	NUB-7, LAN-5, IMR-32 and SK-N-BE(2)	2–8 days	0–100 µM * * Significantly inhibited proliferation in the range of 5–10 µM	↑ p53 translocation from cytoplasm to nucleus ↑ p21^WAF−1/CIP−1^	↑ G1-, G2/M-, and S-phase arrest	[42]
Honokiol	Neuro-2a (mouse cell line) and NB41A3	72 h	2.5, 5, 10, 20, 30, 40, 50, 60, 80, 100 µM LC_50_: 63.3 µM		↑ Sub-G1-phase arrest	[39]
Prenyl hydroxy-coumarins	Neuro-2a (mouse cell line)	24, 48, 72 h	6.25–200 µg/mL		↑ Sub-G1-phase arrest	[43]
Resveratrol	B103 (rat cell line)	48 h	5–20 µM IC_50_: 17.86 µM	↓ Cyclin D1	↑ G1-phase arrest	[40]
Resveratrol	B65 (rat dopaminergic cell line)	24 h	25, 50, 100 µM	↓ pAkt ↓ Cyclin D, E, A ↓ CDK2 ↑ p53 ↑ NFκβ	↑ S-phase arrest	[41]
Resveratrol	NUB-7, LAN-5, IMR-32 and SK-N-BE(2)	2–8 days	25–160 µM	↑ p53 translocation from cytoplasm to nucleus ↑ p21^WAF−1/CIP−1^	↑ G1-, G2/M-, and S-phase arrest	[53]

* ↑ denotes increase of biomarker, while ↓ denotes decrease.

### 3.3. Mitochondrial and ER-Stress-Related Apoptotic Pathways

**Figure 4:**
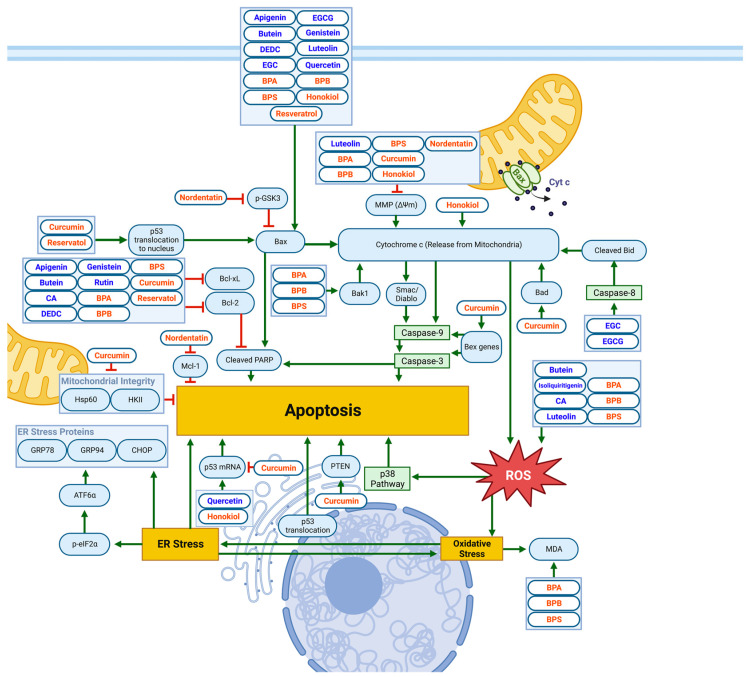
Schematic diagram demonstrating the apoptotic effects of flavonoids (blue) and non-flavonoid polyphenols (orange) on NB cell lines via mitochondrial or ER/oxidative-stress-related pathways. Elevated Bax/Bcl-2 ratio, increased PARP cleavage, loss of MMP and cytochrome C release, and ROS generation all contribute to apoptotic cell death. Specific biomarkers can be viewed in Table 3. Created with BioRender.com.

Results of several studies have also identified Mitochondrial and ER-Stress-Related Apoptotic Pathways, as demonstrated in Figure 4 and Table 3.

#### 3.3.1. Bcl-2-Family Proteins

The relative amounts of mitochondrial protein subfamilies of Bcl-2 (including antiapoptotic Bcl-xL) and pro-apoptotic Bcl-2 associated X-protein (Bax) act as apoptosis triggers. Specifically, a high Bax/Bcl-2 ratio leads to cytochrome c release by the mitochondria [54]. In turn, this causes the activation of apoptotic caspases-9 and -3. Moreover, an increased Bax/Bcl-2 ratio leads to poly (ADP-ribose) polymerase (PARP) cleavage. Flavonoids apigenin, butein, DEDC, genistein, luteolin, EGC, EGCG, and quercetin increase the amount of Bax protein in NB [27,30,32,33,55,56,57,58]. Similar effects on Bax are attributed to the non-flavonoid polyphenols bisphenol-A (BPA), bisphenol-B (BPB), bisphenol-S (BPS), honokiol, and resveratrol [39,42,59]. The flavonoids apigenin, butein, carnosic acid (CA), DEDC, genistein, and rutin and the non-flavonoid polyphenols BPA, BPB, BPS, curcumin, and resveratrol decrease the amount of both Bcl-2 and Bcl-xL, contributing to both cytochrome c release and PARP cleavage in NB [27,32,36,37,39,40,55,56,59,60,61]. The findings in another study indicate that oleacein demonstrates tumor-suppressive effects against SH-SY5Y neuroblastoma cells. Oleacein blocked the S phase of the cell cycle, induced apoptosis via the upregulation of both Bax/Bcl-2 ratio and p53 expressions, and increased STAT3 phosphorylation [62]. Another antiapoptotic protein in the Bcl family IS Mcl-1, whose expression in SH-SY5Y cells is notably reduced by Nordentatin, a coumarin derivative isolated from *Clausena harmandiana*. A reduction in Mcl-1 concentration results in Bax activation. This leads to caspase-3 cleavage and the inhibition of the migratory protein MMP-9 via the glycogen synthase kinase-3 (GSK-3) pathway, ultimately inhibiting the proliferation and migration of NB cells [63].

#### 3.3.2. PARP Cleavage

The proteolytic cleavage of the enzyme PARP is upregulated by caspase-3 and Bax, reducing the inhibition of DNase activity and increasing DNA fragmentation during apoptosis [64,65]. A significant impact on numerous signaling molecules was observed after the administration of isoflavone lupiwighteone in NB cells. Specifically, lupiwighteone inhibited NB-cell growth, induced G2/M phase arrest, and increased apoptosis via the caspase-dependent mitochondrial pathway; these effects were accompanied by decreased cyclin B1/D1 and CDK 1/2/4/6, the effects on MMP, increased ROS, Bax, cytochrome c, cleaved caspase-9 and -3, cleaved PARP, decreased Bcl-2, and activated Nrf2/ARE signaling [66]. Moreover, a potent anti-cancer capacity has recently been attributed to dichloromethane extract from *Scrophularia orientalis* L., a traditional Chinese medicinal plant, which induced cell death. At the same time, these effects were associated with aberrant calcium signaling, leading to mitochondria-related apoptosis, as demonstrated by intracellular Ca^2+^ release, the opening of the mitochondrial permeability transition pore, increased caspase-3, and PARP cleavage [67].

#### 3.3.3. MMP and Cytochrome C

The mitochondrial membrane potential (MMP) generated by proton pumps on the mitochondrial membrane drives the proton gradient exploited in ATP synthesis during oxidative phosphorylation [68]. The loss of MMP imposes a loss of cell viability. Studies involving luteolin, BPS, BPA, BPS, curcumin, and honokiol found the downregulation of MMP to significantly contribute to apoptosis in NB by enhancing cytochrome C release and the release of apoptosis-inducing factor (AIF), an apoptotic protease [39,57,58,59,69]. As with ECG and EGCG, activated caspase-3 can also indirectly cleave BH3-interacting-domain-death agonist (Bid) via the activation of caspase-8, inducing Bid cleavage into truncated-Bid (tBid) and, subsequently, cytochrome c release [37,38,70]. Other studies suggest the role of tBid in the pathway, as it mobilizes cytochrome c across the outer mitochondrial membrane [71]. In NB cells treated with resveratrol, the loss of MMP associated with cytochrome c release induced Smac/Diablo release from the mitochondria, promoting caspase-9 release [61].

#### 3.3.4. Oxidative and ER Stress

The release of cytochrome C by the mitochondria upregulates the generation of reactive oxygen species (ROS), which results in oxidative stress [59]. Oxidative stress contributes to ER stress and vice versa. In addition, ER stress inhibits protein synthesis, eventually leading to apoptosis. The flavonoids butein, CA, isoliquiritigenin, and luteolin and the non-flavonoid polyphenols BPA, BPB, and BPS directly increase the generation of ROS [26,57,59,60,72]. In the luteolin-specific pathway, ROS-associated ER stress leads to eukaryotic Initiation Factor 2α (eIF2α) phosphorylation and, subsequently, activating transcription factor 6α (ATF6α) cleavage. The ATF6α cleavage is correlated with the expression of the ER-stress-related proteins CHOP, GRP94, and GRP78 [57]. Neuroblastoma cells treated with BPA, BPB, and BPS showed elevated malondialdehyde (MDA) levels, reflecting severe oxidative damage [59]. In another study, carnosic acid (CA) showed cytotoxic potential against SH-SY5Y neuroblastoma cells. The CA suppressed methylglyoxal-induced nitrosative and oxidative stress by triggering the PI3K/Akt/Nrf2 signaling pathway. Furthermore, the CA stimulated antioxidant enzymes in neuroblastoma cells through Nrf2-transcription-factor activation [73]. Using the same cells, 3,4-dihydroxybenzalacetone or caffeic acid phenethyl ester induced oxidized-protein-mediated ER stress. In addition, both compounds increased the expression of LC3-II, an autophagy marker, and decreased 4-phenylbutyric acid, a chaperone that reduces ER stress [74]. Graham et al. showed that resveratrol elevated ER stress and the cytotoxic effects of glycolytic inhibition in neuroblastoma cell lines. Analyzing the mechanism of action, the authors described the downregulation of Akt (by increasing PP1α activity) in signaling pathways independent of SIRT1. Moreover, resveratrol initiated both caspase-3- and calpain-mediated apoptosis [75].

#### 3.3.5. p53 and p38

The ROS activates the p38 pathway, as observed in NB cell lines treated by CA [60]. Consequently, p38 increases apoptotic signals, eventually leading to apoptosis. As observed in NB cells, quercetin and resveratrol increase p53 translocation from the cytosol to the nucleus [29]. Furthermore, p53 also played a role in honokiol-triggered autophagic apoptosis via an intrinsic mitochondrion-dependent mechanism [76]. A study investigating curcumin-mediated *Bex*-gene induction found a molecular association between p53 activation and *Bex*-gene induction. Reintroducing all the endogenous anti-cancer *Bex* genes directly contributes to NB-cell death via the activation of the intrinsic apoptotic pathway [65].

**Table 3:** Results of in vitro studies of polyphenols’ apoptotic effects on NB cell lines via mitochondrial apoptotic pathways and/or endoplasmic reticulum stress *.

Compound	Cell Line	Incubation Period	Concentration(s)	Biomarker Changes	Reference
Flavonoids					
Apigenin	SH-SY5Y	24 h	50 µM	↑ Bax ↓ Bcl-2 ↑ Cytochrome *c* release ↑ Caspase-3, -9	[27]
Apigenin	NUB-7 and LAN-5	24 h	10, 50, 100, 150, 200 µM IC_50_: 35 μM in NUB-7 IC_50_: 22 μM in LAN-5	↑ Bax ↑ PARP cleavage ↑ Caspase-3	[33]
Butein	Neuro2a (mouse cell line)	24 and 48 h	6.25, 12.5, 25, 50, 100 μM IC_50_: 6.25 µM, 24 h	↑ Bax ↓ Bcl-2 ↑ Caspase-3 ↑ PARP cleavage ↑ ROS	[55]
CA (carnosic acid, rosemary phenolic compound)	IMR-32	24 h	5, 10, 20, 30, 40 µM IC_50_: 30 µM	↑ Caspase-3, -9 ↑ PARP cleavage ↓ Bcl-2 ↑ ROS ↑ p38 activation	[60]
CA	SH-SY5Y	1 h	0.2–2 μM	↑ Nitrosative and oxidative stress ↑ PI3K/Akt/Nrf2 signaling ↑ Nrf2 expression	[73]
DEDC	SH-SY5Y	24 h	7.5 µg/mL	↑ Bax ↓ Bcl-2	[32]
3,4-dihydroxybenzalacetone and caffeic acid phenethyl ester	SH-SY5Y	4 and 8 h	10 and 20 μM	↑ LC3-II ↓ 4-Phenylbutyric acid (chaperone) ↑ Autophagy	[74]
EGC	SH-SY5Y	24 h	50 µM	↑ Cytochrome c ↑ Caspase-9	[27]
ECGC	SH-SY5Y	24 h	50 µM	↑ Cytochrome c ↑ Caspase-9	[27]
Genistein	SK-N-DZ	24 h	10 µM	↑ Caspase-3, -9 ↑ Bax/Bcl-2 ratio ↓ Bcl-2 with only genistein Complete Bcl-2 knockdown with combination treatment (with Bcl-2 siRNA plasmid vector)	[56]
Genistein	SH-SY5Y	24 h	100 µM	↑ Bax ↓ Bcl-2 ↑ Cytochrome *c* ↑ Caspase-3, -9	[27]
Isoliquiritigenin	IMR-32 and SK-N-BE(2)	24 h	5–200 μM	↑ ROS level	[72]
Luteolin	Neuro-2a (mouse cell line)	24 h	1–50 µM IC_50_: 10 µM	↑ ER: CHOP, GRP94, GRP78 ↑ ATF6α cleavage ↑ eIF2α phosphorylation ↑ ROS ↓ MMP ↑ Bax ↑ cytochrome *c*	[57]
Quercetin	Neuro2a (mouse cell line)	24 h	10, 20, 40, 80, 120 μM IC_50_: 40 µM	↑ Caspase-3, -9 ↑ p53 mRNA ↑ Bax ↑ Cytochrome c	[30]
Rutin	LAN-5	24 h	0, 25, 50, 100 μM	↓ Bcl-2 expression ↑ Bax/Bcl-2 ratio	[36]
**Non-Flavonoid Polyphenols**					
Bisphenol A (BPA) Bisphenol B (BPB) Bisphenol S (BPS)	IMR-32 (male) and SK-N-SH (female)	24 h	BPA and xBPS: IMR-32: 1, 10, 100 nM IMR-32 and SK-N-SH: 1, 10, 100 μM BPB: IMR-32: 1, 10 nM IMR-32 and SK-N-SH: 100 nM 1, 10, 100 μM	↑ Caspase-3 ↑ Bak1 ↑ Bax ↑ Cytochrome c ↓ Bcl-2 ↓ MMP ↑ ROS ↑ MDA	[59]
Curcumin	NUB-7, LAN-5, IMR-32 and SK-N-BE(2)	2–8 days	25–160 µM	↑ p53 translocation ↑ Bax	[42]
Curcumin	GI-L-IN, HTLA-230, SH-SY5Y, LAN5, SK-NBE2c, and IMR-32	18–72 h	0.1–25 µM	↑ Cytochrome C	[38]
Curcumin	SK-N-SH	24 h	8, 16, 32 µM	↑ Caspase-3 ↑ ROS ↑ PARP cleavage ↓ p53 ↓ Bcl-2 ↓ MMP ↑ Cytochrome C	[37]
Curcumin	LAN-5	3, 5, 24 h	5, 10, 15, 20 µM	↓ Hsp60 ↓ HK-II	[77]
Curcumin	Neuro-2a (mouse cell line)	24 h	10, 25, 50 µM	↑ Caspase-3, -9 ↑ ROS ↑ PARP cleavage ↑ p53 ↑ *Bex* genes	[65]
Honokiol	Neuro-2a (mouse cell line) and NB41A3	72 h	2.5, 5, 10, 20, 30, 40, 50, 60, 80, and 100 µM LC_50_: 63.3 µM	↑ Bax ↑ Cytochrome-C ↓ MMP ↑ Caspase-3, -6, -9	[39]
Honokiol	Neuro-2a (mouse cell line) and NB41A3	24, 48, 72 h	50 µM	↑ p53 ↑ Cytochrome-C ↑ Autophagy ↑ Caspase-3 ↑ LC3-II	[76]
Nordentatin	SH-SY5Y	24, 48, 72 h	1, 10, 100 µM	↓ GSK-3 phosphorylation ↓ Mcl-1 ↓ MMP-9 ↑ Caspase-3	[63]
Oleacein	SH-SY5Y	6 and 24 h	10 and 25 μM	↑ Bax/Bcl-2 ratio ↑ p53 ↑ STAT phosphorylation	[62]
Resveratrol	SK-N-AS, NGP, and SH-SY5Y	48 h	IC_50_: SK-N-A: 70 µM/L NGP: 120 µM/L SH-SY5Y: 100 µM/L	↑ Caspase-3, -9 ↑ Cytochrome-C ↑ Smac/Diablo ↓ Bcl-2	[61]
Resveratrol	B103 (rat cell line)	48 h	5–20 µM IC_50_: 17.86 µM	↓ Bcl-2 ↓ Bcl-xL ↓ Mcl-1 ↑ Caspase-3, -9	[40]
Resveratrol	NUB-7, LAN-5, IMR-32 and SK-N-BE(2)	2–8 days	25–160 µM	↑ Bax ↑ p53 translocation	[42]
Resveratrol	K-N-SH, SH-SY5Y, SK-N-Be2, SMS-KCNR, and NB1691	8 h	10–100 μM	↑ Cell death ↑ Caspase-3 ↑ ER stress ↓ Akt ↑ PP1α	[75]
**Plant extracts**					
Kaffir lime leaf (contains alkaloid, flavonoid, terpenoid, tannin, and saponin compounds)	UKF-NB3, IMR-5 and SK-N-AS		IC_50_: UKF-NB3: 18.9 µg/mL IMR-5: 6.4 µg/mL SK-N-AS: 9.4 µg/mL		[78]
Juniperus communis L. Berry (contains 13 flavonoid glycosides and 2 phenolic acids)	SH-SY5Y	12, 36, 48 h	10 µg/mL	↑ p53	[79]

* ↑ denotes increase of biomarker, while ↓ denotes decrease.

### 3.4. Receptor-Mediated Apoptotic Pathway

**Figure 5:**
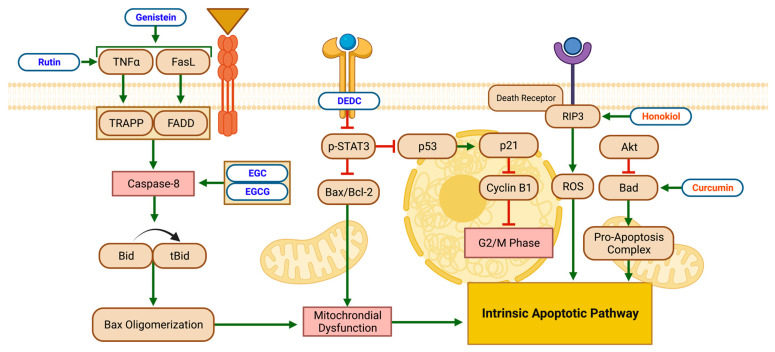
Schematic diagram demonstrating the apoptotic effects of flavonoids (blue) and non-flavonoid polyphenols (orange) on NB cell lines via receptor-mediated pathways. Intrinsic apoptotic pathways were induced via mitochondrial dysfunction (influenced by Bax-protein levels) and pro-apoptotic complexes (due to Bad-protein increase). Specific biomarkers can be viewed in Table 4. Created with BioRender.com.

Receptor-mediated pathways primarily trigger apoptosis and inhibit cell-cycle progression through various mechanisms, which are outlined in Figure 5 and Table 4. The mechanisms of flavonoid and non-flavonoid polyphenols’ actions match those of the previously discussed pathways.

The flavonoid DEDC decreases the expression of phospho-STAT3 in a ROS-mediated manner in NB cells [32]. The downregulation of phospho-STAT3 leads to the upregulation of p53 and p21, downregulating Cyclin B1, resulting in cell-cycle arrest and apoptosis in the G2/M phase [32]. 

The flavonoids genistein and rutin have similar mechanisms, leading to apoptotic effects. Rutin increases NB’s necrosis factor α (TNF-α) secretion [36]. Similarly, genistein increased TNF-α and Fas ligand (FasL), TRADD, and FADD in NB [56]. The upregulation of TNF and FasL lead to upregulated TNF receptor type 1-associated DEATH domain protein (TRADD), and Fas-associated DEATH domain protein (FADD) activates caspase-8, which cleaves Bid to tBid. Increased levels of tBid encourage conformational changes in Bax, forming oligomer channels, which promote cytochrome c release. The release of tBid activates caspase-3, leading to nuclear-DNA fragmentation and apoptosis. In addition, both studies found a decrease in antiapoptotic Bcl-2, an increase in pro-apoptotic Bax and, therefore, an overall increase in the Bax:Bcl-2 ratio, which triggers the mitochondrial pathway of apoptosis by releasing cytochrome c in NB. This mechanism is also present when EGC and EGCG increase the activation of caspase-8 and the proteolytic cleavage of Bid to tBid, and Bax oligomerization leads to apoptosis [27].

Similarly, EGC and/or EGCG showed significant effects through the induction of both mitochondria-mediated (as evidenced by increased Bax and decreased Bcl-2) and receptor-mediated apoptotic pathways (demonstrated via the activation of caspase-8 and the cleavage of Bid into tBid) in two NB-cell lines. However, the effects of the EGC and EGCG on apoptosis were accompanied by decreased oncogenic miRNAs and increased tumor-suppressor miRNAs. Furthermore, the authors concluded that the overexpression of tumor-suppressor miRNA-7-1 increases the efficacy of EGCG in apoptosis induction, suggesting the potential of this combination therapy in NB [52].

The non-flavonoid polyphenol curcumin decreases heat shock protein-60 (Hsp60) and hexokinase II (HK-II) levels and increases Bcl-2 associated agonist of cell death (Bad), phosphatase and tensin homolog (PTEN), and ROS in NB cells [77]. The mitochondrial protein Bad increases mitochondrial membrane permeability, promoting cytochrome c release and apoptosis via an intrinsic apoptotic pathway [77]. Phosphatase and tensin homolog is associated with the (PI3K)/Akt pathway—a “highly oncogenic pro-survival” signaling pathway. The PTEN antagonizes the (PI3K)/Akt pathway, leading to ROS-mediated apoptosis [77]. Honokiol, another non-flavonoid polyphenol, demonstrated upregulation of ROS, resulting in increased receptor-interacting protein kinase 3 (RIP3), a critical regulator of programmed necrosis [80].

Resveratrol showed its capacity to increase caspase-3, resulting in an inhibited cell cycle in NB cells [41]. Activated caspase-3 plays a role in cell maintenance and survival through the cleaving of multiple structural and regulatory proteins. Additionally, activated caspase-3 contributes to mitochondrial apoptotic processes, such as releasing cytochrome c and Bax translocation [81].

## 4. Overview of Findings

### 4.1. Potent Findings and Treatment Mechanisms

Currently, the approaches that utilize natural compounds, including flavonoids, in treating NB cell lines, can be characterized into four primary mechanisms of treatment: the induction of apoptosis via mitochondrial ER pathways, receptor-mediated apoptosis, calpain-driven apoptosis, and anti-proliferative mechanisms.

Calpain-dependent apoptosis is one mechanism that can be exploited in NB treatment. The flavonoids apigenin, EGC, and EGCG exert apoptotic effects at the lowest concentration (50 µM). However, the finding of calpain-driven apoptosis in NB cell lines via the application of flavonoids was presented only by Das et al. (2006) [27]. Apigenin is rich in dried parsley, with a concentration of 45,035 μg/g, while EGC and EGCG, which are both types of catechin, can be found in green tea [82]. In addition, EGC was found at a concentration of 1160.6 μg/mL, while the concentration of EGCG was 542.6 μg/mL, when green tea was brewed for 80 min at 50 °C, suggesting green tea as a potent source of flavonoids [83]. Green tea is mainly consumed in Asian countries, such as China, Japan, Korea, and India; this indicates a relationship between green-tea consumption and the relatively low frequency of NB in all of Asia, although this proposition requires further research [84,85].

In the studies reviewed here, curcumin, quercetin, and resveratrol are the most heavily researched polyphenol compounds in relation to NB treatment, and exert the most potent effects. In two studies, curcumin decreased cell growth and induced G1-, G2/M-, and S-phase arrest at concentrations of 0.1–25 µM and 5–10 µM, respectively [38,42]. Pure turmeric powder has one of the highest concentrations of curcumin, at around 3.14% by weight; thus, it is a potentially significant source of curcumin in cancer-treatment supplementation [86]. Quercetin decreased levels of Bcl-w and Cyclin-CDK at an IC50 of 40 µM; in another study, quercetin inhibited cell growth at an IC50 of 10 µM [30,31]. However, despite these promising IC_50_ values, which suggest high potency, the studies were conducted using mouse cell lines rather than human-NB-cell lines. Regarding natural sources, multiple berries are rich in quercetin, such as cranberries, with concentrations of 83–121 mg/kg. Furthermore boiling blueberries (1 g/10 mL) for 4 min yields a quercetin concentration of 1138.4 mg/mL [87,88]. Isoliquiritigenin, another flavonoid, is mainly found in licorice (*Glycyrrhiza uralensis*), at a concentration of 0.45 mg/g [89]. The anti-proliferative effect of isoliquiritigenin is also promising, with an IC_50_ of 25.4 µM [35]. In other studies, isoliquiritigenin had apoptotic effects on prostate cancer cells, melanoma, leukemia, ovarian cancer, lung cancer, and colon cancer [90].

Another notable anti-NB mechanism is the regulation of p53 expression. Most of the papers reviewed showed an increase in the tumor-suppressor gene, resulting in cell death. However, a study conducted by Ye et al. (2021) demonstrated that treatment with curcumin decreased p53 levels but increased NB apoptosis [37]. This suggests an apoptosis mechanism that is not p53-dependent, or the possibility of p53 exerting dual effects [91].

The most studied form of anti-NB mechanism was the induction of apoptosis via ER and/or mitochondria-related apoptotic mechanisms, including, but not limited to, cytochrome c release, Bax/Bcl-2, ROS generation, and p53 expression. Butein induced apoptosis via increased Bax/Bcl-2 ratio, PARP cleavage, and ROS at a low IC_50_ of 6.5 µM [55]. Luteolin also demonstrated notable apoptotic effects, at an IC_50_ of 10 µM; it increased cytochrome c release, Bax/Bcl-2, ER-stress proteins, ATF6α cleavage, and eIF2α phosphorylation [57]. Onion leaves are among the many naturally occurring sources of luteolin, with a measured content level of 391.0 mg/kg, making them excellent candidates for supplementary NB therapies [92]. Other naturally occurring sources abundant in luteolin include carrots, parsley, apple skin, and broccoli [93].

Many other flavonoids also induced apoptosis, although the studies on butein and luteolin reported the lowest IC_50_ values for flavonoids exerting apoptotic effects via mitochondrial/ER pathways. One of the more widely studied compounds is curcumin: two studies reported curcumin’s involvement in mitochondrial/ER pathways to induce apoptosis [37,38]. Three studies using resveratrol to treat NB cell lines reported apoptosis via mitochondrial/ER pathways [40,42,61]. Rahman et al. (2012) reported the lowest IC_50_ (17.86 µM) of these three studies. Notably, this result was obtained by treating a rat cell line rather than a human cell line [40].

Honokiol, a constituent of the traditional Chinese medicinal plant, *Magnolia officinalis*, has been shown to pass through the blood–brain barrier (BBB) without affecting normal brain cells [39,94]. Studies have demonstrated its induction of autophagic apoptosis in NB cells via an intrinsic mitochondrion-dependent pathway and its involvement in the ROS-mediated upregulation of RIP3 protein [39,76,80]. With a growing body of research to support its potential, honokiol is a significant candidate for use as therapy for high-risk NB.

Apoptosis through receptor-mediated pathways involves specific receptors to induce an apoptotic cascade. Of the studies found, none mentioned a specific IC_50_ value. Curcumin, EGC, and EGCG are polyphenol compounds demonstrating notable apoptotic effects via receptor-mediated pathways. The main apoptotic mechanisms reported were increased TNF-α, caspase-8 activation, tBid generation, adaptor protein FADD, and Bax oligomerization [27,77]. As in the studies reporting apoptosis via mitochondrial/ER pathways, the Bax/Bcl-2 ratio is also prominent in receptor-mediated apoptosis. Many receptor-mediated apoptotic pathways depend on mitochondrial apoptotic pathways, further underlining the vital role of the mitochondria in the apoptosis of treated NB lines. Moreover, the prevalence of Bax and Bcl-2 as biomarkers was expected, as the pro-apoptotic protein Bax commits the cell to a mitochondrial suicide pathway and is considered one of the main targets of anti-cancer interventions [95].

### 4.2. Plant Extracts

Plant extracts often contain polyphenols and are investigated for their anti-cancer properties. A paper by Tunjung et al. (2015) investigated Kaffir lime leaf (which contains alkaloid, flavonoid, terpenoid, tannin, and saponin compounds) against three NB cell lines. The IC_50_ values were as follows: UKF-NB3 cell line, 18.9 µg/mL; IMR-5 cell lines, 6.4 µg/mL; and SK-N-AS cell lines, 9.4 µg/mL [78]. Another paper, by Lantto et al. (2016), investigated the extract of *Juniperus communis* L. Berry (containing 13 flavonoid glycosides, 2 phenolic acids) by treating the NB cell line SH-SY5Y with 10 µg/mL. The extract increased the expression of the tumor-suppressor gene p53 [79]. More studies are needed to determine whether combinations of polyphenols (as seen in plant extracts) work synergistically against NB, or whether these summations have no significant effects.

### 4.3. In Vivo Studies Involving NB

Although the IC_50_ values of polyphenols demonstrate considerable potency, a vehicle for effective flavonoid delivery remains unclear. Firstly, it is not feasible to translate in vitro concentrations into dosages for clinical use. Compared to in vitro studies, fewer in vivo studies demonstrate polyphenols’ anti-NB abilities. However, researchers investigating compounds in live mouse models have demonstrated noteworthy results (Table 5). These studies consider three alternate flavonoid-delivery methods: oral, intraperitoneal, or peritumor injection. Resveratrol is the most frequently studied polyphenol, with three papers supporting its in vivo efficacy [61,96,97]. All three of the proposed delivery methods proved effective; one study, which utilized oral delivery, found an 80% decrease in tumor volume, despite the low bioavailability of resveratrol [61]. The five studies outlined in Table 5 highlight an area that needs future study; data from mice models can aid the clinical implementation of natural compounds in the treatment of NB.

## 5. Key Considerations and Challenges

### 5.1. Limitations of Polyphenol Use

Polyphenols have the potential to exert adverse effects in certain circumstances. Polyphenols’ toxicity in normal cells involves their pro-oxidant behavior in the presence of redox-active metals, which is unusual given their antioxidant activity. Pro-oxidant polyphenols have a negative effect on DNA, proteins, and lipids. Toxicity can also form tumors in normal cells, where a few phenolic acids, which are examples of polyphenols, have tumor-inducing properties [98].

The ingestion of large doses of polyphenols is associated with carcinogenesis, thyroid toxicity, and the disruption of the bioavailability of pharmaceuticals. Increased polyphenol levels have also been linked to increased kidney tumors in mice and rats [99]. Notably, the inability to mimic in vivo conditions in in vitro studies limits our understanding of polyphenol toxicity in human health [20].

### 5.2. Insights into Flavonoid-Delivery Mechanisms

A physical mechanism of polyphenol delivery is difficult to achieve. Specifically, the delivery of flavonoids is associated with several limitations due to their low water solubility and low bioavailability [100]. Therefore, many delivery methods have been used and tested to increase flavonoids’ bioavailability when they are consumed orally. Two prominent strategies explored in the literature are nanotechnology and encapsulation methods, as they have shown the ability to overcome barriers to flavonoid delivery. Nanotechnological strategies encompass a wide variety of therapeutic delivery methods. Much of the research into nano-based delivery explores ways of increasing herbal drugs’ bioavailability and protection against gastrointestinal (GI) deterioration. Nano-structural (such as protein, carbohydrate and/or lipid-based carriers) and nanoparticle delivery systems for flavonoids have presented increased bioavailability due to their ability to prevent GI degradation, increasing flavonoids’ absorption rate into the bloodstream [101,102].

Additionally, nanocrystal methods are used, as the small size of the particle or compound increases its dissolution rate (effects vary based on the surface area available for interactions with their target), increasing the bioavailability of compounds upon delivery [101]. The nano-micellar delivery of flavonoids has also been studied for its enhanced bioavailability properties. These micelles can be developed via the self-assembly of amphiphilic molecules in an appropriate liquid medium to improve and maintain targeted delivery.

Encapsulation methods involve trapping a compound of interest (in this paper, flavonoids) in sealed miniature nano-capsules. They have been studied in the literature for their ability to improve flavonoid distribution, their solubility, and their stability in the body—as with nanotechnological strategies—by protecting the compound from oxidation and degradation upon oral consumption [101].

The presence of the BBB also affects the bioavailability of flavonoids. It plays an essential role in protecting the brain through its selective permeability of substances entering the brain—such as essential nutrients and hormones—and elimination of toxins [103].

The current literature on flavonoids and the BBB suggests that the permeability of a flavonoid depends on the compound’s properties and how they interact with the properties of the BBB. Lipid solubility is one of the primary properties of flavonoids that affect their permeability into the BBB [104,105]. For the compound to cross the BBB, there are two primary transport mechanisms: passive diffusion (as studies have found that lipophilic flavonoids demonstrate increased transportation rates with increased concentrations) and efflux transporters [105]. Efflux transporters, such as P-glycoprotein, play a role in controlling the transport of xenobiotic agents. Research suggests that efflux transporters may play a role in the permeability rates of flavonoids by limiting their bioavailability and distribution across the BBB [106].

One study evaluating the transport of flavonoids across the BBB found that the flavonoids genistein and isoliquiritigenin had higher permeation than rutin and quercetin [105]. Notably, it also found that combining a flavonoid with weaker permeability, such as rutin, with verapamil (a P-glycoprotein inhibitor) can help to enhance its permeability across the BBB.

Zhenzhu et al. (2022) underline the potential for extracellular vesicles (EVs) to penetrate brain tissue and the BBB, specifically for treating neurodegenerative (ND) diseases. Extracellular vesicles are naturally produced by many cell types and include subtypes such as exosomes, microvesicles, apoptotic bodies, and oncosomes, all ranging in size and application [107]. Extracellular vesicles have been studied in the delivery of polyphenols to treat neurodegenerative diseases. The flavonoids studied include curcumin, quercetin, and resveratrol [108,109,110].

### 5.3. Clinical Trials Investigating Therapeutic Use of Polyphenols

While conducting searches for clinical-trial data across databases such as ClinicalTrials.gov and the Cochrane Central Register of Controlled Trials (CENTRAL), relatively very few clinical studies on neuroblastoma treatments were found, and none of these investigate polyphenols as potential treatments, despite promising in vitro research, as well as clinical trials on other types of cancer. The majority of the completed clinical studies regarding interventional treatments for neuroblastoma feature limitations in their characteristics, as well as presenting serious and non-serious adverse effects on their participants [111,112,113,114,115]. This demonstrates the need to explore and investigate other treatment options, among which polyphenols have been proposed based on current in vitro research, as well as clinical studies on the effects of polyphenols on other types of cancer.

Upon expanding the search from “neuroblastoma” to “cancer” and polyphenols on clinical-trial databases, a handful of clinical trials with published data were found. The most common cancers studied were prostate, breast, oral, and colon cancers. Of the polyphenols discussed in this paper, curcumin, EGCG, resveratrol, quercetin, and apigenin have been submitted to 71, 39, 17, 14, 1, and 0 clinical trials, respectively [21]. For this section, 13 completed and published clinical studies were selected from the previously mentioned clinical databases.

Overall, many of these clinical trials presented promising results that suggested the potential of polyphenols as cancer treatments, with the outcomes outlined in Table 6. Safety and tolerability were demonstrated across these studies [21], with most reporting no adverse effects of the polyphenols tested on their participants. The trial results were found to be positively correlated with the results of in vitro studies—however, the results should be interpreted with caution due to the limited statistically significant results and study characteristics.

Research regarding childhood cancer and polyphenols is rarely performed. One dissertation featured a pilot study, entitled “Purple Grape Juice in Improving Vascular Health in Childhood Cancer Survivors”, with 24 participants, to evaluate whether purple grape juice can reduce oxidative stress and improve the vascular health of survivors of childhood cancer who are in early stages of cardiovascular disease [128]. The results showed no improvements in endothelial function and no significant improvements in oxidative stress and inflammation biomarkers.

Across the clinical studies discussed, many limitations arose from the study characteristics and methodologies, which included:

1. Non-blinded studies [116,117,122].
2. The effects of physiological parameters on the results [117,121], including varying rates of disease progression in sample populations [126].
3. Small sample sizes [117,119,120,121,122,123,124,125,126,127,128].
4. Potential measurement errors [123], or invalidated assays [119].
5. Low bioavailability due to the poor absorption of polyphenols [125,127].
6. The short duration of trials [118,121,122,123,124,126,128].
7. Reliance on a few select endpoints without other indicators of disease progression [118].

Despite the limitations in the currently available data, polyphenols are still under consideration as candidates for cancer treatment due to their potential as less toxic treatments [21]. Of the clinical trials discussed, a few of those that presented significant results involved trials with strong study characteristics, primarily randomization, placebo control, and double masking [118,121], suggesting potential for future studies and clinical applications. Therefore, the future of cancer and polyphenol research will need to focus on improving study characteristics, testing different methods of delivering polyphenols, and expanding to other types of cancer to properly assess polyphenols’ anti-cancer properties and how to best deliver them to patients. Furthermore, these clinical studies, alongside in vitro studies, support the investigation of the application of polyphenols as treatments for neuroblastoma.

## 6. Conclusions

Polyphenols are natural compounds synthesized by plants, and they can be divided into flavonoids and non-flavonoids. Polyphenols have shown promising results in both in vitro and in vivo studies of their use against neuroblastoma, a cancer that predominantly affects children. The anti-proliferative effects of polyphenols include arresting the cell cycle, decreasing bcl-w and cyclin-CDK levels, and increasing p53 levels. Polyphenols can also affect apoptotic mechanisms, including apoptosis, via mitochondrial ER pathways, through which polyphenols can promote cytochrome c release and increase the Bax/Bcl-2 ratio and ROS levels. Another apoptotic mechanism that polyphenols utilize is receptor-mediated apoptosis, which is dependent on the mitochondrial apoptotic pathway and involves TNF-α and caspase-8 activation and Bax oligomerization. Calpain-driven apoptosis was only reported by a single paper, making it a potential topic for future research. The use of flavonoid delivery, including a solution to the question of how to increase its bioavailability orally and across the blood–brain barrier, as the physical mechanism of delivery, is difficult to achieve. There is inadequate in vivo evidence to suggest that in vitro success would translate into clinical studies, and all the apoptotic mechanisms should be researched further, despite the encouraging results so far.

## Figures and Tables

**Table 1:** Results of in vitro studies of polyphenols’ apoptotic effects on NB cell lines via a calpain-dependent pathway *.

Compound	Cell Line	Incubation Period	Concentration(s)	Biomarker Changes	Reference
Flavonoids					
Apigenin	SH-SY5Y	24 h	50 µM	↑ Intracellular free [Ca^2+^] ↑ Calpain activation ↑ Caspase-12, -3 ↑ CAD	[27]
EGC	SH-SY5Y	24 h	50 µM	↑ Intracellular free [Ca^2+^] ↑ Calpain activation ↑ Cytoskeletal protein degradation ↑ Caspase-3 ↑ CAD	[27]
EGCG	SH-SY5Y	24 h	50 µM	↑ Intracellular free [Ca^2+^] ↑ Calpain activation ↑ Cytoskeletal protein degradation ↑ Caspase-3 ↑ CAD	[27]
Genistein	SH-SY5Y	24 h	100 µM	↑ Intracellular free [Ca^2+^] ↑ Calpain activation ↑ Caspase-12	[27]

* ↑ denotes increase of biomarker, while ↓ denotes decrease.

**Table 4:** Results of in vitro studies of polyphenols’ apoptotic effects on NB cell lines via receptor-mediated pathways *.

Compound	Cell Line	Incubation Period	Concentration(s)	Biomarker Changes	Reference
Flavonoids					
DEDC	SH-SY5Y	24 h	7.5 µg/mL	↓ Phosphor-STAT3 expression (ROS mediated)	[27]
Genistein	SK-N-DZ	24 h	10 µM	↑ TNF-α ↑ FasL ↑ TRADD ↑ FADD	[27]
EGC	SH-SY5Y	24 h	50 µM	↑ Caspase-8 activation ↑ Proteolytic cleavage of Bid to tBid ↑ Bax oligomerization	[27]
EGCG	SH-SY5Y	24 h	100 µM	↑ Caspase-8 activation ↑ Proteolytic cleavage of Bid to tBid ↑ Bax oligomerization	[27]
Rutin	LAN-5	24 h	25, 50, 100 μM	↑ TNF-α secretion	[36]
**Non-Flavonoid Polyphenols**					
Curcumin	LAN-5	3, 5, 24 h	5, 10, 15, 20 µM	↑ Bad ↑ PTEN ↑ ROS	[77]
Honokiol	Neuro-2a (mouse cell line)	30, 60, 120 µM	24, 48, 72 h	↑ RIP3 ↑ ROS	[80]

* ↑ denotes increase of biomarker, while ↓ denotes decrease.

**Table 5:** Results of in vivo studies of polyphenols’ apoptotic effects on NB cell lines *.

Compound	Cell Line	Incubation Period	Dose(s)	Effect(s)	Reference
Flavonoids					
Didymin	Subcutaneous injection of 2 × 10^6^ SMS-KCNR NB-cell suspensions into athymic nude mice	8 weeks	2 mg/kg (oral gavage on alternate days)	↓ CD31 (angiogenesis marker) ↓ ki67 (proliferation marker) ↓ N-Myc (NB oncogenic marker) ↓ Tumor mass	[34]
Apigenin	Scapular injection of 5 × 10^6^ NUB-7 NB-cell suspensions into nonobese diabetic/severe combined immunodeficient mice	1 week	25 mg/kg (intraperitoneal injection daily)	↓ Tumor mass (~50% less) ↑ Apoptotic fraction	[33]
**Non-Flavonoid Polyphenols**					
Curcumin	Orthotopic injection of 1.5 × 10^6^ GI-LI-N NB-cell suspensions into athymic nude mice	4 weeks	17.5 mg/kg (intravenous injection twice a week)	↓ Tumor-growth rate	[38]
Resveratrol	Subcutaneous injection of 3 × 10^6^ SK-N-AS NB-cell suspension into athymic nude mice	5 weeks OR 16 days	50 mg/kg (oral gavage daily) OR 20 mg (peritumor injection 5 times)	↓ Tumor volume (~80% less)	[61]
Resveratrol	Subcutaneous injection of 1 × 10^6^ Neuro-2a-cell suspension into A/J mice	4 weeks	40 mg/kg (intraperitoneal injection daily)	↓ Tumor-growth rate ↑ Long-term survival rate (~70%)	[96]
Resveratrol	Subcutaneous injection of 2 × 10^6^ NXS2 NB-cell suspension injected into A/J mice	2 weeks	20 mg (peritumor injection twice a week)	↓ Tumor-growth rate ↑ Long-term survival rate (~61%)	[97]

* ↑ denotes increase, while ↓ denotes decrease.

**Table 6:** Primary details from selected clinical studies on cancer and polyphenols.

Cancer Type (No. of Selected Studies)	Source of Polyphenols	Noted Outcomes *
Prostate Cancer (5)	Green Tea and Pomegranate	Four studies produced statistically significant results, including decreased nuclear NFκB staining [116], decreased prostate-specific antigen (PSA) levels [117,118], and prolongation of PSA doubling time [119]. Another study also presented decreased PSA in study participants undertaking a trial polyphenol treatment, but this did not reach statistical significance [120].
Breast Cancer (3)	Green Tea and Pomegranate	Statistically significant results, such as a decrease in serum hepatocyte growth factor (HGF) [121]. Non-statistically significant results included decreased serum vascular endothelial growth factor (VEGF) [121], as well as decreased serum HGF, in a different study [122]. Another study analyzed the effects of pomegranate juice on hormonal biomarkers of breast cancer risk [123]. Although the results presented statistically significant reductions in serum estrone and testosterone levels in women of normal weight, these results were not observed in overweight/obese women.
Oral Cancer	Green Tea and Pomegranate	One study investigated the effects of green tea, and the results presented a non-statistically significant downregulation of angiogenic stromal VEGF [124]. Another study investigated the effects of curcumin, with the results presenting a statistically significant reduction in inflammatory-cytokine concentrations in salivary cells [125].
Colorectal Cancers	Green Tea and Pomegranate	One study investigated green tea, which produced a non-statistically-significant reduction in percentage change in rectal aberrant crypt foci (ACF) number (compared to baseline measurements). These ACFs are generally seen as precursors of colorectal cancers [126]. Another study’s results showed no correlation between the levels of metabolites and the degree of differentiation of adenocarcinomas when investigating pomegranate extract [127].

* Only some results were selected from across the studies to provide the primary outcome(s). For the complete results of each study, please refer to their citations in the references.

## Data Availability

No new data were created or analyzed in this study.

## Abbreviations

Akt	Serine–threonine kinase
AIF	Apoptosis-inducing factor
ATF	Activating transcription factor
Bax	Bcl-2 associated X-protein
Bad	Bcl-2 associated agonist of cell death
BBB	Blood–brain barrier
Bcl	B-cell lymphoma
Bid	BH3 interacting-domain death agonist
BP	Bisphenol
Ca^2+^	Calcium ion
CAD	Caspase-activated DNase
CDK	Cyclin-dependent kinase
CHOP	C/EBP homologous protein
DEDC	2-(cis-1,2-dihydroxy-4-oxo-cyclohex-5-enyl)-5,7-dihydroxy-chromone
EGCG	Epigallocatechin gallate
EGC	Epigallocatechin
eIF	Eukaryotic initiation factor
ER	Endoplasmic reticulum
ERK	Extracellular signal-regulated kinase
FADD	Fas-associated DEATH domain protein
FasL	Fas ligand
GI	Gastrointestinal
GRP	Glucose-regulated protein
GSK	Glycogen synthase kinase
HSP	Heat-shock protein
JNK	c-Jun N-terminal kinase
MAPK	Mitogen activated protein kinase
MDA	Malondialdehyde
MMP	Mitochondrial membrane potential
NB	Neuroblastoma
NF-κB	Nuclear Factor κappa of B cells
PARP	Poly (ADP-Ribose) polymerase
PI3K	Phosphoinositide 3-kinases
PTEN	Phosphatase and tensin homolog
RIP	Receptor-interacting protein
RKIP	Raf-1 kinase inhibitor protein
ROS	Reactive oxygen species
tBid	Truncated Bid
TNF	Tumor necrosis factor
TRADD	Tumor necrosis factor receptor type 1-associated DEATH domain
